# Influence of Live Attenuated *Salmonella* Vaccines on Cecal Microbiome Composition and Microbiota Abundances in Young Broiler Chickens

**DOI:** 10.3390/vaccines11061116

**Published:** 2023-06-19

**Authors:** Wilfred Michael Lyimu, Samson Leta, Nadia Everaert, Jan Paeshuyse

**Affiliations:** 1 Laboratory of Host-Pathogen Interactions in Livestock, Division of Animal and Human Health Engineering, Department of Biosystems, KU Leuven, 3001 Leuven, Belgium; wilfredmichael.lyimu@kuleuven.be (W.M.L.); samsonleta.regassa@kuleuven.be (S.L.); 2 Department of Biomedical Sciences, College of Veterinary Medicine and Agriculture, Addis Ababa University, Bishoftu P.O. Box 34, Ethiopia; 3 The Nutrition and Animal Microbiota Ecosystems Laboratory, Division of Animal and Human Health Engineering, Department of Biosystems, KU Leuven, 3001 Leuven, Belgium; nadia.everaert@kuleuven.be

**Keywords:** *Salmonella*, live vaccine, poultry, cecum, microbiota, cytokine, 16S rRNA sequencing

## Abstract

Salmonellosis is a global food safety challenge caused by *Salmonella*, a gram-negative bacterium of zoonotic importance. Poultry is considered a major reservoir for the pathogen, and humans are exposed through consumption of raw or undercooked products derived from them. Prophylaxis of *Salmonella* in poultry farms generally mainly involves biosecurity measures, flock testing and culling, use of antibiotics, and vaccination programs. For decades, the use of antibiotics has been a common practice to limit poultry contamination with important pathogenic bacteria such as *Salmonella* at the farm level. However, due to an increasing prevalence of resistance, non-therapeutic use of antibiotics in animal production has been banned in many parts of the world. This has prompted the search for non-antimicrobial alternatives. Live vaccines are among the developed and currently used methods for *Salmonella* control. However, their mechanism of action, particularly the effect they might have on commensal gut microbiota, is not well understood. In this study, three different commercial live attenuated *Salmonella* vaccines (AviPro^®^ Salmonella Vac T, AviPro^®^ Salmonella DUO, and AviPro^®^ Salmonella Vac E) were used to orally vaccinate broiler chickens, and cecal contents were collected for microbiomes analysis by 16S rRNA next generation sequencing. Quantitative real-time PCR (qPCR) was used to study the cecal immune-related genes expression in the treatment groups, while *Salmonella*-specific antibodies were analyzed from sera and cecal extracts by enzyme-linked immunosorbent assay (ELISA). We show that vaccination with live attenuated *Salmonella* vaccines had a significant influence on the variability of the broiler cecal microbiota (*p* = 0.016). Furthermore, the vaccines AviPro^®^ Salmonella Vac T and AviPro^®^ Salmonella DUO, but not AviPro^®^ Salmonella Vac E, had a significant effect (*p* = 0.024) on microbiota composition. This suggests that the live vaccine type used can differently alter the microbiota profiles, driving the gut colonization resistance and immune responses to pathogenic bacteria, and might impact the overall chicken health and productivity. Further investigation is, however, required to confirm this.

## 1. Introduction

*Salmonella* is an important pathogenic bacterium in the poultry industry, public health, and human food safety worldwide. It is implicated in both animal and human salmonellosis cases. Unlike their typhoidal counterparts (*Salmonella enterica* serovar Gallinarum and *Salmonella enterica* serovar Pullorum) which are host-specific, non-typhoidal Salmonella (*Salmonella enterica* serovar Enteritidis, *Salmonella enterica* serovar Typhimurium, *Salmonella enterica* serovar Infantis) can infect a range of hosts and are of zoonotic importance, being associated with human food poisoning cases. Infection with *Salmonella* in humans occurs upon consumption of raw or undercooked poultry products, mostly meat and eggs, contaminated with the pathogen [1]. In young chicks, whose immunity is still poorly developed and whose gut microbiome is immature, infection with non-typhoidal *Salmonella enterica* can be systemic and deadly. Healthy older chickens, however, remain asymptomatic upon infection, silently propagating the pathogen in the flock, contaminating the products thereof and increasing the incidences of human salmonellosis [2,3,4,5]. Poultry is thus an important reservoir for non-typhoidal *Salmonella* and control measures which limit their contamination with this pathogen at the farm level would greatly contribute to a reduction in human non-typhoidal salmonellosis cases [6].

Generally, the most commonly used prophylaxes in poultry farms against *Salmonella* involve biosecurity measures, flock testing and culling, use of antibiotics, and vaccination programs. For decades, the chemoprophylactic use of antibiotics as feed additives has been widely practiced and with considerable success in limiting intestinal pathogenic bacterial infections [7,8]. The increasing global problem of antimicrobial resistance, however, has led to the banning of antibiotics as growth promoters in poultry production in most countries. In Europe, this has been in effect since 2006 under the Commission regulation (EC) No 1177/2006 [9]. Consequently, non-antibiotic alternatives for the control of important pathogenic bacteria such as *Salmonella* in poultry are urgently needed. Prebiotics, probiotics, bacteriophages, phytobiotics and vaccines are among the developed non-antibiotic prophylactic measures. The mechanism of action of most of these new anti-*Salmonella* strategies are, however, not fully understood [10,11].

Nevertheless, most of the developed anti-*Salmonella* alternatives are administered orally to effectively reach the gut, which is the main route for *Salmonella* entry in chicken. Successful colonization of the chicken gut by non-typhoidal *Salmonella* involves induction of inflammation by invasion of the epithelial cells. This also generates metabolites such as tetrathionate, which acts as a terminal electron acceptor needed for ethanolamine and 1,2-propane diol utilization under anaerobic conditions [12]. From this, *Salmonella* gains a competitive advantage over the commensal gut bacteria. Consequently, this leads to an imbalanced gut microbiota, allowing for colonization of the chicken gut by *Salmonella*. Thus, control approaches that lead to a strengthened or balanced protective microbiota in the chicken gut are essential to limiting *Salmonella* contamination level in chickens. The commensal gut microbiota play an important role in protecting chickens against *Salmonella* via mechanisms such as modulation of the host immune responses, colonization exclusion, short chain fatty acids (SCFA) release from the metabolism of non-fermentable carbon sources and antimicrobial peptides (such as bacteriocins) production. The latter two are, respectively, bacteriostatic and bactericidal to *Salmonella* [10,13].

Live bacteria prophylactics, involving the use of attenuated *Salmonella* vaccines and live bacteria with protective benefits (probiotics) to the chicken gut are commonly used methods in modern poultry farming. The varying efficacy of live *Salmonella* vaccines in protecting chickens against the challenge of wild-type strains has been reported [14]. Nevertheless, vaccination is still considered the most efficient approach to control *Salmonella* contamination in chickens at the farm level [15]. Both inactivated and live attenuated *Salmonella* vaccines for poultry are commercially available and used in poultry farms. Whereas inactivated vaccines mainly induce humoral immunity, live attenuated *Salmonella* vaccines can trigger both cellular and humoral immune responses, offering more protection in poultry [14]. Cellular responses are also associated with an enhanced expression of interferon gamma (IFNγ), interleukin 8 (IL-8) and inducible nitric oxide synthase (iNOS) while downregulating interleukin 1β (IL-1β) [16]. 

The effect of live attenuated *Salmonella* vaccines on the poultry gut microbiota is not well established. A recent study by Park et al. [17], using *Salmonella* Typhimurium live attenuated vaccine candidates, reported compositional changes but not overall relative abundance in gut microbiota. To our knowledge, reports on other live attenuated *Salmonella* vaccine types, particularly multivalent vaccines, are not available. In this study, we used three commercial live attenuated *Salmonella* vaccine strains for *S.* Typhimurium (AviPro^®^ Salmonella Vac T), *S*. Enteritidis (AviPro^®^ Salmonella Vac E) and *S.* Typhimurium + *S.* Enteritidis (AviPro^®^ Salmonella DUO) to study their influence on cecal microbiota profiles. The cecal innate immune genes expressions and *Salmonella*-specific humoral responses (IgG and IgA) were also investigated in both vaccinated and unvaccinated chickens.

## 2. Materials and Methods

### 2.1. Experimental Animals Description

A total of 16 day-old male Ross-308 broiler chicks were obtained from a commercial supplier (Belgabroed nv, Merksplas, Belgium). The chicks used for this study were pre-vaccinated for Newcastle disease by the supplier via spray with Nobilis^®^ ND C2 (MSD Animal Health, Madison, NJ, USA), a live Newcastle disease vaccine. The use of animals for this study was evaluated and approved by the KU Leuven Ethical Committee for Animal Research, project number P040/2020.

### 2.2. Animal Handling Procedures and Housing

The experiment was carried out at TRANSfarm, the test facility of KU Leuven, located in Lovenjoel, Belgium. The chicks were housed in 4 pens (4 chicks/pen) with the floor covered by wood shavings. A starter diet and drinking water were provided *ad libitum* in a room with standard heating and light-dark cycles. 

### 2.3. Experimental Setup and Treatments

Day-old broiler chicks were divided into four treatment groups: *S.* Typhimurium (AviPro^®^ Salmonella Vac T), *S.* Enteritidis (AviPro^®^ Salmonella Vac E), *S.* Typhimurium + *S.* Enteritidis (AviPro^®^ Salmonella DUO) and the control group, each consisting of four chicks. The live attenuated *Salmonella* vaccines (AviPro^®^ Salmonella Vac T, AviPro^®^ Salmonella Vac E and AviPro^®^ Salmonella DUO) used in this study were all obtained from Elanco Europe Ltd. (Bartley Wood Business Park, Hook, UK). At 2 days of age, cloacal swabs were collected from all chicks to check for the presence of *Salmonella* contamination prior to administration of the treatments. Then, each group received 0.5 mL of either a live attenuated *Salmonella* vaccine or sterile normal saline (for the control) by oral gavage. On day 14 post-vaccination (DPV14, 16 days post hatch), the experiment was terminated, and samples were collected. 

### 2.4. Sample Collection and Processing

DPV14 (16 days post hatch), chickens were euthanized by cervical dislocation, dissected, ceca and cecal contents were aseptically collected into sterile 2 mL cryovials (SARSTEDT, Nümbrecht, Germany), immediately placed on dry ice for transportation and then stored at −80 °C until use. One chick from the *S.* Enteritidis (AviPro^®^ Salmonella Vac E) group could, however, not be sampled as it died four days after vaccination and was thus excluded from further analysis. The specific cause of the chick’s death was not clear, as the bird did not show any visible physical indications of disease or deformation. Nevertheless, failure of adaptation to the rapid changes that occur during the first week of the chick’s life, where their immunity is still immature (transportation stress, feed, water, environmental, and microbiological changes), is the suspected cause. Prior to humane killing, whole blood was withdrawn from each chicken’s brachial wing vein into BD Vacutainer™ SST™ serum separator tubes (Fisher Scientific, Hampton, NH, USA) for serum collection. Serum was prepared by centrifugation at 2000× *g* for 10 min at room temperature, collecting a clear supernatant into new tubes and storing at −20 °C until needed. The sampling in this study was intended to capture the cecal immune responses in the function of live *Salmonella* vaccines and the vaccines interaction with commensal microbiota. Thus, the collection of samples was performed towards the broilers gut microbiota stabilizing period, which is suggested to be 14–21 days post hatch [18].

### 2.5. Total Tissue RNA Extraction and Complementary DNA (cDNA) Synthesis

Total RNA was isolated from cecal tissues using the TRIzol™ (Invitrogen™, Waltham, MA, USA) method, following the manufacturer’s instructions. Briefly, approximately 80 mg cecal tissues were homogenized in TRIzol™ (Invitrogen) for 1 min at 6800 rpm, in the presence of sterile 2 mm Zirconia Beads (BioSpec, Bartlesville, OK, USA), using the Precellys^®^ Evolution tissue homogenizer (Bertin instruments, Montigny-le-Bretonneux, France). Chloroform (Avantor, Radnor, PA, USA) was added to tissue homogenates, followed by centrifugation for 15 min at 12,000× *g*, 4 °C to separate the aqueous from the organic phase. Total RNA was precipitated from the aqueous phase by addition of isopropanol (Carl Roth, Karlsruhe, Schoemperlenstraße, Germany), followed by centrifugation for 10 min at 12,000× *g*, 4 °C. The RNA pellet was washed and resuspended in 75% ethanol (VWR, Geldenaaksebaan, Leuven, Belgium), vortexed briefly and centrifuged for 5 min at 7500× *g*, 4 °C. The supernatant was discarded with a micropipettor, and the RNA pellet allowed to air-dry for 10 min. The RNA pellet was finally solubilized via resuspension in 40 µL RNase-free water through gentle pipetting. The concentration and purity (A260/A280 ratio) of the RNA samples were checked using a SimpliNano^TM^ spectrophotometer (BioChrom, Hill Road Holliston, MA, USA), then stored at −80 °C until needed.

The cDNA was synthesized from 200 ng cecal total tissue RNA per sample, using oligo (dT) primers (Promega, Madison, Wisconsin, MA, USA) and the Maxima H minus reverse transcriptase enzyme (ThermoFisher, Waltham, MA, USA), following the manufacturer’s protocol. In brief, the cDNA synthesis was performed at 50 °C for 30 min in the presence of 200U Maxima H minus reverse transcriptase (ThermoFisher Scientific) and 20U RiboLock RNase inhibitor (ThermoFisher Scientific). The reaction was then terminated by heating at 85 °C, and samples were stored at −20 °C until use.

### 2.6. qPCR

A probe-based qPCR was performed using the GoTaq probe two-step-qPCR system (Promega), according to the manufacturer’s recommendations. The PCR reactions consisted of 10 µL GoTaq probe master mix (2X), 0.4 µL each primer per target (200 nM), 0.4 µL hydrolysis probe (200 nM), cDNA (70 ng per sample) and nuclease-free water to a final 20 µL reaction volume. The reactions were prepared in MicroAmp^TM^ Fast Optical 96-well reaction plates and sealed with MicroAmp^TM^ optical adhesive films (ThermoFisher Scientific). The PCR run was performed in the Applied Biosystems StepOne Plus (ThermoFisher Scientific) using the standard cycling conditions; 50 °C for 2 min, 95 °C for 2 min, then followed by 40 cycles of 95 °C for 15 s and 60 °C for 1 min. The primer pairs and hydrolysis probes used for this study are given in Table 1. The design of primers and probes and the checking of their parameters were performed using the integrated DNA technology (IDT) OligoAnalyzer^TM^ tool (IDT, Leuven, Belgium) and were ordered from the same manufacturer.

### 2.7. ELISA

An overnight culture of a live attenuated *Salmonella* vaccine strain (AviPro^®^ Salmonella DUO) was pelleted at 10,000× *g* (4 °C) for 5 min and washed twice with PBS at the same speed, each time for 1 min. The bacterial pellet was resuspended in PBS and absorbance (O.D_600_) was adjusted to 1, using a nanospectrophotometer (Westburg Life Sciences, Leusden-zuid, Utrecht, The Netherlands). This was then distributed in 96-well F-bottom, clear, high binding microplates (Greiner Bio-One, Kremsmünster, Austria) at 50 µL per well as a *Salmonella* vaccine coating solution. Plates were covered with lids, sealed with parafilm to prevent evaporation and incubated overnight at 4 °C to allow for coating. The coating solution was discarded and each well blocked with 50 µL blocking buffer (PBS + 2% BSA + 1% goat serum) for 1.5 h at room temperature. The blocking buffer was then discarded and each well rinsed twice with washing buffer (PBS + 0.05% Tween-20). Prior to use, the standard chicken sera and cecal extract samples were thawed and spun down at 11,000× *g* (1 min, room temperature). The standard samples were derived from 53 week-old Isa Brown layer chickens, obtained from Evap Proefbedrijf Pluimveehouderij (Provincie Antwerpen, Belgium) and were fully vaccinated orally with the AviPro^®^ Salmonella DUO live attenuated vaccine. For cecal extracts preparation, 0.5 g cecal contents were homogenized in 500 µL extraction buffer (PBS + 0.02% sodium azide) by continuously vortexing for 15 min. The mixture was then centrifuged at 7000× *g* for 20 min (4 °C) and clear supernatant collected. Prior to use as standards, chicken sera and cecal extracts were tested for high absorbances (antibodies) by ELISA and then aliquoted into 200 µL and stored at −20 °C until needed. Fifty microliters of the diluted standard and test samples were pipetted into each well of the coated plates as primary antibodies and incubated at room temperature for 1 h. The unbound antibodies were washed by rinsing 3 times with washing buffer, followed by thorough blotting on paper towel. Fifty microliters horse-radish peroxidase (HRP)-conjugated rabbit anti-chicken IgG (EMD Millipore AP162P) or goat anti-chicken IgA (Bio-Rad, Hercules, CA, USA), were added as secondary antibodies (1:10,000, in blocking buffer) and plates incubated at room temperature for 30 min. Unbound secondary antibodies were washed by rinsing 4 times and thorough blotting of the plates on paper towel. To each well, 50 µL 3,3′5,5′-tetramethylbenzidine (TMB) developing substrate (ThermoFisher Scientific) was pipetted and plates incubated at room temperature for 10 min. The enzymatic reaction was finally stopped using 50 µL 0.18 M sulfuric acid and absorbance immediately measured at 450 nm using the Victor3 microplate reader (Perkin Elmer, Waltham, MA, USA).

The antibody units were assigned to standard and test samples as previously described by Miura et al. [27], with some modifications. Briefly, an aliquot of the standard serum sample was thawed, centrifuged at 11,000× *g* for 1 min and diluted in 2-fold steps from 1:100 to 1:51,200 in blocking buffer. For cecal extracts, the thawed aliquot was centrifuged for 1 min at 7000× *g*, then diluted in ten 2-fold steps from 1:10 to 1:10,240. The serially diluted standards were applied on ELISA plates as primary antibodies, except for two wells per set of serially diluted standard which were left as blank wells and assigned the reciprocal number of dilution 0. The relation between reciprocal number of dilution and absorbance (OD_450_) was approximated using MyCurveFit (https://mycurvefit.com/, accessed on 15 March 2023) [28], an online 4-parameter hyperbolic curve fitting software. As a quality check, only standard samples giving R^2^ ≥ 0.994 were used for further analyses of the test samples. The constants of the hyperbolic curve fit equation were used to assign antibody units to the standard as reciprocal of the dilution giving an OD_450_ = 1. After determining the antibody units of the standard samples, the number was used for all samples tested by ELISA against that standard. Such a reference standard was then used on each ELISA plate to make a standard curve. To this end, standard serum and cecal extract aliquots were thawed and used to prepare ten 2-fold dilution steps in duplicates serially, starting with a dilution of 20 antibody units (serum) and 2 antibody units (cecal extracts). The serially diluted reference standards and test samples were applied to the assigned wells on ELISA plates as primary antibodies and four were left as blank wells (antibody units assigned as 0). The absorbance (OD_450_) values obtained were fitted to a 4-parameter standard curve (Antibody units = a[{(a − OD_450_)/(OD_450_ − d)}˄(1/b)]), using an online 4-parameter hyperbolic curve software (MyCurveFit). The generated standard curves were then used to calculate antibody units in test samples from their measured absorbance values. The antibody units in undiluted samples were finally determined based on the dilution factor for each sample.

### 2.8. Total Genomic DNA (gDNA) Isolation for 16S rRNA Sequencing

Genomic DNA was isolated from the chicken cecal contents using the QIAamp^®^ Fast DNA Stool Mini kit (Qiagen, Hilden, Germany), according to the manufacturer’s protocol. The concentration and purity of the isolated genomic DNA from cecal contents were checked using the SimpliNano^TM^ spectrophotometer (BioChrom), and the A260/A280 ratios were within the recommended range (1.8–2.0) for pure DNA. The DNA samples were then stored at −20 °C until use.

### 2.9. Sample Preparation and 16S rRNA Next Generation Sequencing

DNA concentration measurements were performed using the Qubit double-stranded DNA (dsDNA) high-sensitivity assay kit and Qubit^TM^ 3.0 fluorometer (ThermoFisher Scientific). To normalize for concentration, all samples were diluted to the lowest measured concentration. The V4 region of the 16S rRNA gene was amplified as previously described [29], with few modifications. Briefly, 10 µL of the template DNA, 5 µL Phusion HF Buffer (5X), 0.5 µL dNTPs mix (10 nM), 0.25 µL Phusion HF DNA polymerase (2 U/µL), 2.5 µL forward primer (5 µM), 2.5 µL reverse primer (5 µM) and 4.25 µL nuclease free water were used at a final 25 µL reaction volume per sample. Both the Phusion HF Buffer (5X) and Phusion HF DNA polymerase were obtained from New England BioLabs^®^ Inc. (Ipswich, MA, USA). For PCR amplification of the V4 region, unique barcoded primers 515F (5′-AATGATACGGCGACCACCGAGATCTACAC NNNNNNNN TATGGTAATT GT*GTGCCAGCMGCCGCGGTAA*-3′) and 806R (5′-CAAGCAGAAGACGGCATACGAGAT NNNNNNNN AGTCAGTCAG CC *GGACTACH VGGGTWTCTAAT*-3′) were used. The following PCR program was used; initial denaturation (98 °C, 30 s), 24 cycles of denaturation (98 °C, 10 s), annealing (55 °C, 30 s) and extension (72 °C, 30 s), and final extension (72 °C, 5 min). The PCR products were purified using AMPure XP beads (Illumina, San Diego, CA, USA), following the manufacturer’s instructions. The purification and correct amplicon (385 bp) were confirmed by electrophoresis on a 1% agarose gel in tris-base acetic acid EDTA (TAE) buffer. Equimolar (8 nM) amplicons at a final volume 10 µL for each sample were then pooled into a sterile 1.5 mL microcentrifuge tube (SARSTEDT), in Illumina resuspension buffer. The pooled samples were denatured and diluted to 1.5 pM. Finally, 350 µL of the 16S library (1.5 pM), 150 µL genomic library prepared using the Illumina DNA prep (20060060) and 15 µL of PhiX (1.5 pM) were combined. This combination was loaded on the MiniSeq Mid output (300 cycles) reagent cartridge (Illumina) and custom primers were added to the reagent cartridge as follows; 16.5 µL of 10 µM Read1.515F (5′-TATGGTAATTGTGTGCCAGCMGCCGCGGTAA-3′) was added to position 24; 18.3 µL of 10 µM Read2.806R (5′-AGTCAGTCAGCCGGACTACHVGGGTWTCTAAT-3′) added to position 25; 24.6 µL of 10 µM Index1.806R (5′-ATTAGAWACCCBDGTAGTCCGGCTGACTGACT-3′) and 25.3 µL of 10 µM Index2.515F (5′-TTACCGCGGCKGCTGGCACACAATTACCATA-3′) were added to position 28. During sequencing, paired-end reads (2 × 150 bp) were generated. The sequencing was performed at the Laboratory of Gene Technology (KU Leuven, Belgium).

### 2.10. Data Analysis

The demultiplexed files acquired from the sequencing platform were processed using the LotuS pipeline (version 1.62.1) [30], with default parameters. In brief, the pipeline was used for reads1 and reads2 assembly, sequences quality checking, clustering, operational taxonomic units (OTUs) generation, and taxa assignments by Basic Local Alignment Search Tool (BLAST) against the SILVA database (version SILVA 138.1 SSU). The R package phyloseq (version 1.38.0) was used to import and organize the microbiota data. The aggregate rare function from microbiota package (version 1.16.0) was used to combine rare taxa into “others” category. The packages ranacapa (version 0.1.0) and ggplot2 (version 3.3.6) were used to generate the rarefaction curve. The R packages microbiota (version 1.16.0), ggplot2 (version 3.3.6) and hrbrthemes (version 0.8.0) were used for plotting the taxa abundances at genus and phylum levels. The microbiota R package was also used to plot alpha diversity while phyloseq R package was used to plot beta diversity. The adonis2 function from vegan package (version 2.5-7) was used to test if the control and the treatment groups were significantly different from each other via permutational multivariate analysis of variance (PERMANOVA), using distance matrices. All R-based analyses were performed in R software version 4.2.1 [31].

The qPCR data were analyzed using the ΔΔCt (fold change) method on Microsoft Excel (version Microsoft 365) to compute the cecal immune-related genes expression. The data were then further analyzed using the GraphPad Prism 9.0.0 (San Diego, CA, USA) software for statistical testing in different treatment groups, in which two-way ANOVA was used. GraphPad Prism was also used to compare the cecal microbiotas taxa abundances, and antibodies level in different treatment groups. For these purposes, the Kruskal–Wallis test was used and *p* values ≤ 0.05 were considered significant. Data are presented as mean and standard error of the mean (mean ± S.E.M).

## 3. Results

### 3.1. Live Vaccines and Cecal Immune Genes Expression

**Figure 1:**
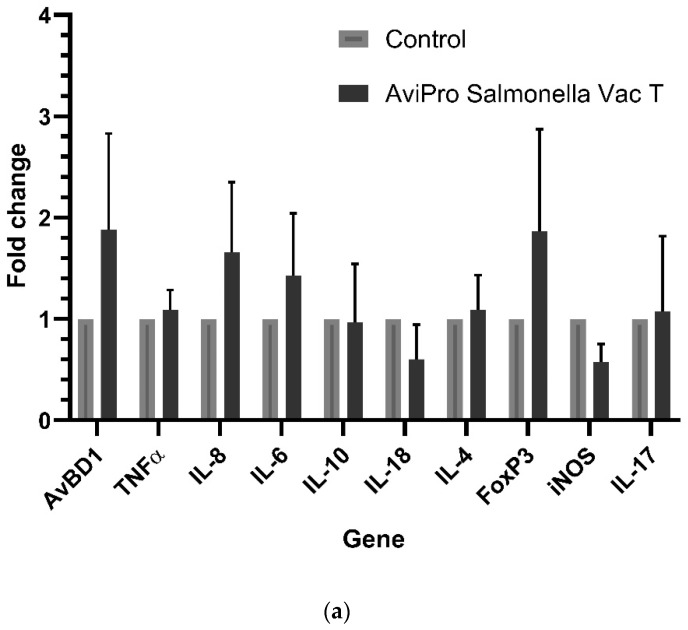

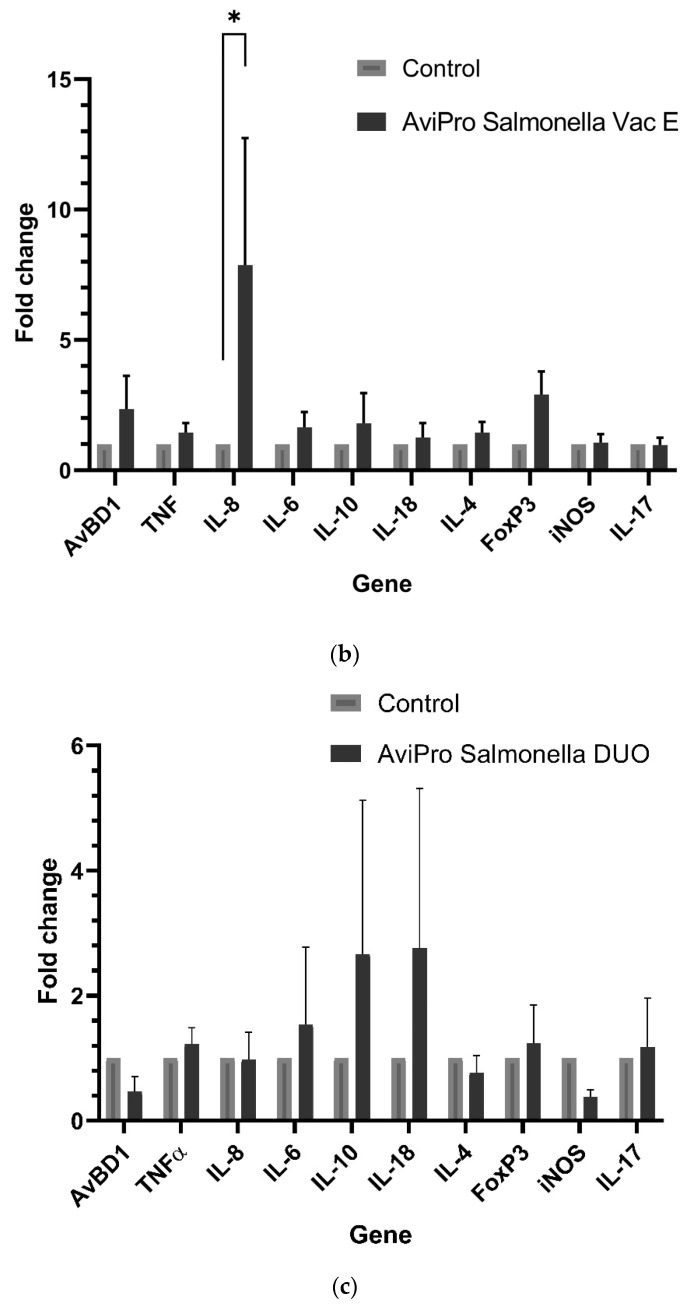
Cecal immune-related genes expression in chicks following an oral exposure to commercial live attenuated *Salmonella* vaccines. IL-8 expression was significantly (*p* = 0.0222) induced in AviPro^®^ Salmonella Vac E vaccinated as compared with the unvaccinated control chickens. Although the expressions of other immune genes were also altered following vaccination with all the vaccines studied here, the observed changes were not significantly different (*p* values > 0.05) from the control level: (**a**) Genes expression after oral vaccination with a live attenuated *Salmonella* Typhimurium vaccine (AviPro^®^ Salmonella Vac T); (**b**) Genes expression after oral vaccination with a live attenuated *Salmonella* Enteritidis vaccine (AviPro^®^ Salmonella Vac E); (**c**) Genes expression after oral vaccination with a live attenuated *Salmonella* DUO vaccine (AviPro^®^ Salmonella DUO). The control group received sterile normal saline, also by oral gavage. AviPro^®^ Salmonella DUO (DUO) = *Salmonella* Typhimurium + *Salmonella* Enteritidis vaccine; AvBD1 = avian β-defensin 1; TNFα = tumor necrotic factor α (also known as LITAF = lipopolysaccharide-induced TNF factor); IL-8 = interleukin 8 (avian chemokine CXCLi2 = a functional homolog of mammalian IL-8); IL-6 = interleukin 6; IL-10 = interleukin 10; IL-18 = interleukin 18; IL-4 = interleukin 4; IL-17A = interleukin 17A; iNOS = inducible nitric oxide synthase; FoxP3 = Forkhead Box P3. Glyceraldehyde 3-phosphate dehydrogenase (GAPDH) was used as a reference (endogenous control) for normalization and analysis of genes expression. Three independent qPCR were performed, each time in duplicates. * = *p* ≤ 0.05. Data are presented as mean ± standard error of the mean (mean ± S.E.M).

To investigate the live *Salmonella* vaccines-induced cecal immune related genes, we compared the expression level of selected inflammatory and regulatory cytokines and chemokines (IL-6, IL-8, IL-10, IL-18, IL-4, IL-17A, and TNFα), avian β-defensin (AvBD1), Forkhead Box P3 (FoxP3) and inducible nitric oxide synthase (iNOS) in vaccinated and unvaccinated chickens 14 days after oral immunization. Our data show that the vaccine AviPro^®^ Salmonella vac T induced a downregulation of IL-18 and iNOS and IL-10 while triggering the expression of IL-6, IL-8, TNFα, IL-17A, IL-4, AvBD1 and FoxP3 (Figure 1a). The expression levels for these genes were, however, not significantly different in vaccinated as compared with unvaccinated controls (*p* values > 0.05). 

Compared with the other two vaccines, AviPro^®^ Salmonella Vac E positively induced the expression of all the cecal immune genes investigated. The genes, with their fold change shown in parentheses, were IL-18 (1.3-fold), IL-10 (1.8-fold), IL-6 (1.6-fold), IL-8 (8-fold), TNFα and IL-4 (1.4-fold), IL-17A and iNOS (1-fold), FoxP3 (3-fold), and AvBD1 (2-fold), as per Figure 1b. Though IL-8 was significantly induced (*p* = 0.0222), the expression of the rest of the genes studied here were not significantly triggered by AviPro^®^ Salmonella Vac E (*p* > 0.05).

Likewise, the vaccine AviPro^®^ Salmonella DUO induced the expression of IL-18 (3-fold), IL-10 (3-fold), IL-6 (2-fold), IL-17A, FoxP3 and TNFα (1.2-fold). Unlike the other two vaccines, however, AviPro^®^ Salmonella DUO induced a downregulation of AvBD1, IL-4 and iNOS, and of unaltered IL-8 compared with unvaccinated chickens (Figure 1c). Despite the observed fold expression changes for the genes, these alterations were not significant (*p* values > 0.05) in vaccinated as compared with unvaccinated controls.

### 3.2. Serum and Cecal Antibodies (IgG and IgA) Quantification 

**Figure 2:**
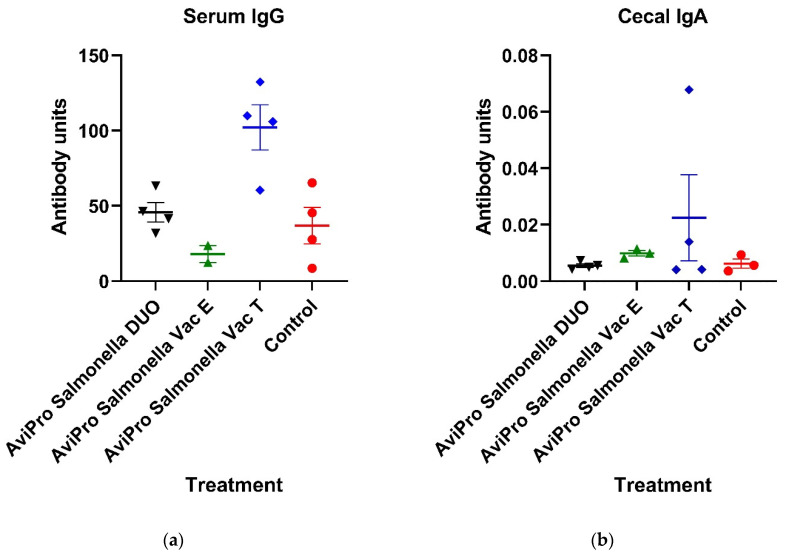
*Salmonella* vaccine-specific antibodies quantification in broiler chickens’ sera (IgG) and cecal contents (IgA). Vaccination with live attenuated *Salmonella* vaccines significantly increase (*p* values > 0.05) neither the serum IgG nor the gut IgA levels in chickens. Chicks were orally administered with a live attenuated *Salmonella* Typhimurium vaccine (AviPro^®^ Salmonella Vac T), *Salmonella* Enteritidis vaccine (AviPro^®^ Salmonella Vac E) or *Salmonella* DUO vaccine (AviPro^®^ Salmonella DUO). The control group received sterile normal saline, also by oral gavage. AviPro^®^ Salmonella DUO = *Salmonella* Typhimurium + *Salmonella* Enteritidis vaccine: (**a**) *Salmonella* vaccine-specific IgG in chicken serum; (**b**) *Salmonella* vaccine-specific IgA in chicken cecal extracts. Both IgG and IgA were quantified by sandwich ELISA and expressed as antibody units. Four chickens were used per treatment, except for the AviPro^®^ Salmonella Vac E in which one chick died four days post immunization and could not be sampled. Additionally, the cecal content collected from one chick in the control group (**b**) was not sufficient for IgA extraction and was excluded from further analysis. The possible reasons for the insufficient cecal content from this chick compared with others could be that either the bird had less feed intake or that the ceca were already emptied some hours before the sampling was performed. Data are presented as mean ± S.E.M.

To study vaccine-induced humoral responses, the quantities of *Salmonella*-specific antibodies (IgG and IgA) were measured in chicken sera and cecal contents, respectively, by ELISA. Our data (Figure 2a) show that IgG in chickens vaccinated with the AviPro^®^ Salmonella Vac T vaccine was about 2-fold higher (*p* = 0.1038) compared with both the AviPro^®^ Salmonella Vac E and AviPro^®^ Salmonella DUO, and unvaccinated controls. The quantity of AviPro^®^ Salmonella Vac E-specific and AviPro^®^ Salmonella DUO-specific IgG antibodies, on the other hand, were low and were not different from the unvaccinated controls. However, all observed differences in IgG levels between vaccinated and control chickens were not statistically significant (*p* values > 0.05).

*Salmonella*-specific IgA antibodies in chicken cecal extracts were generally low in both vaccinated and control chickens (Figure 2b). Furthermore, the antibody levels were not significantly higher in vaccinated as compared with unvaccinated control chickens (*p* values > 0.05).

### 3.3. 16S rRNA Sequencing Depth

**Figure 3:**
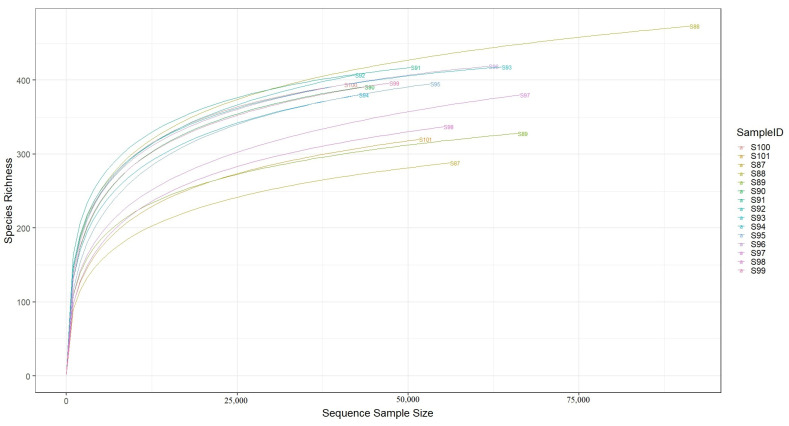
The rarefaction curve reveals that the depth of sequencing was sufficient to cover most of the microbiomes in the samples. Genomic DNA derived from chicken cecal contents was used for 16S rRNA sequencing and microbiome profiling following different treatment under this study. S87, S88, S89 and S90 are the samples derived from the unvaccinated control chickens; S91, S92, S93 and S94 represent the samples from the AviPro^®^ Salmonella Vac T vaccinated chickens; S95, S96, S97 and S98 represent samples from the AviPro^®^ Salmonella DUO vaccine chickens; and S99, S100 and S101 are the samples from the AviPro^®^ Salmonella Vac E vaccinated chickens.

Prior to further analyses for cecal microbiomes, the sequencing depth in different treatments was checked by comparing the sampled sequences derived from the 16S rRNA gene and the rarefaction curves were plotted. As indicated in Figure 3, the identified species increased with the number of sampled 16S rRNA sequences. Furthermore, the plateauing nature of the curves observed here suggests that the depth of our sequencing data was sufficient to cover most of the microbiomes in the samples.

### 3.4. Live Salmonella Vaccine Influence on Cecal Microbiota

**Figure 4:**
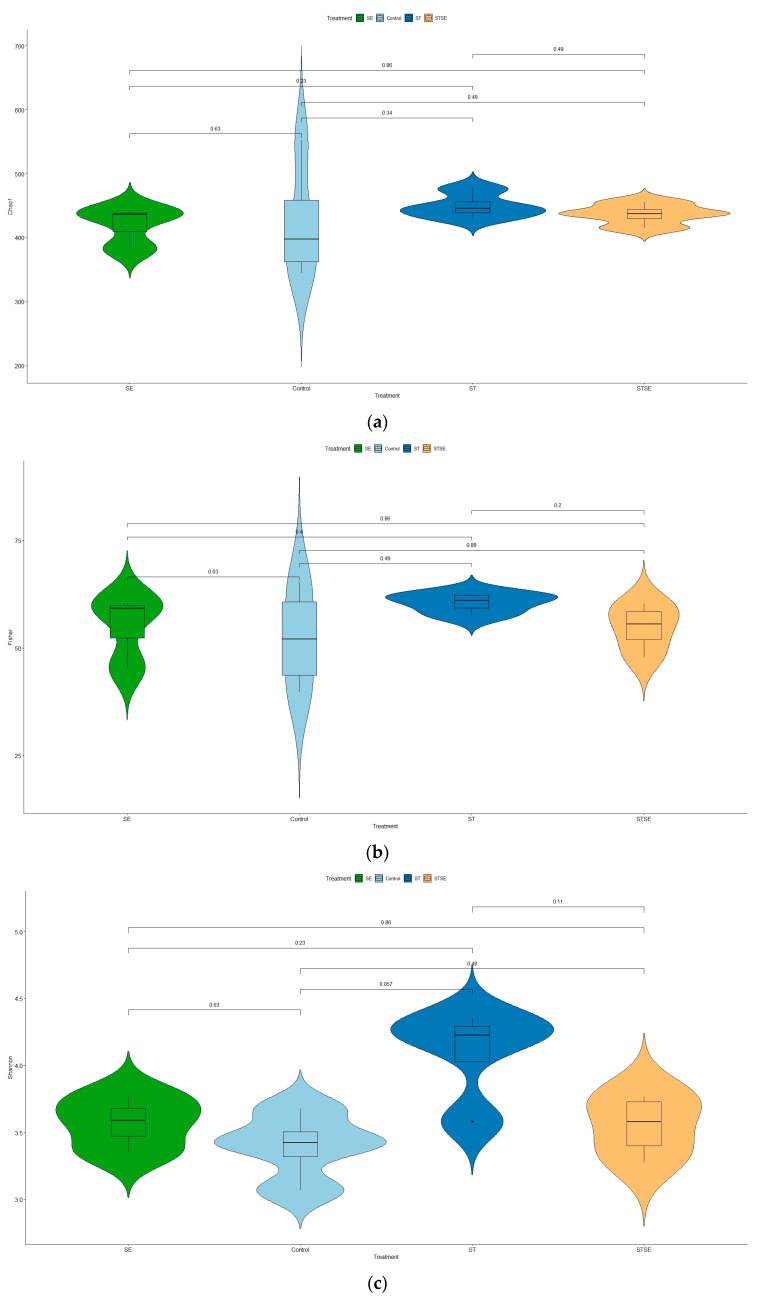
Alpha diversity measures in different treatment groups. No significant variability in all the treatment groups (**a**,**b**), although a marginally significant (*p* = 0.057) homogeneous species distribution was observed in the AviPro^®^ Salmonella Vac T vaccinated chickens, as compared with the control chickens (**c**): (**a**) Chao1 α—diversity estimate plot; (**b**) Fisher α—diversity estimate plot; (**c**) Shannon α—diversity estimate plot. Different diversity indices were used to account for both richness (Chao1, Fisher) and evenness (Shannon) in the samples. The α—diversity estimates were plotted by treatment type (control = unvaccinated; SE = AviPro^®^ Salmonella Vac E; ST = AviPro^®^ Salmonella Vac T; STSE = AviPro^®^ Salmonella DUO).

The influence of live attenuated *Salmonella* vaccines on the chicken cecal microbiota was analyzed using alpha (**α**)-diversity estimates and the beta (β)-diversity measure. Alpha diversity is a measure of species diversity or variability within samples and comprises both the species richness and evenness. In this study, the **α**-diversity estimates were performed using different indices to take into account both richness (Chao1, Fisher) and evenness (Shannon). Our data indicate that there were no significant species diversity in any of the treatment groups (Figure 4a,b). Nevertheless, a marginally significant (*p* = 0.057) homogeneous species distribution was observed in the AviPro^®^ Salmonella Vac T vaccinated chickens, compared with control (Figure 4c).

Subsequently, β-diversity analysis was performed, in which adonis2-based permutation multivariate analysis of variance (PERMANOVA) was used to investigate the influence of vaccination on chicken cecal microbiota.

**Figure 5:**
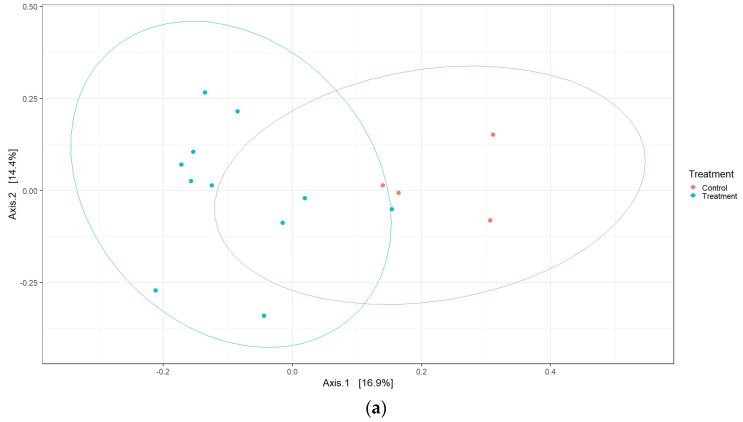

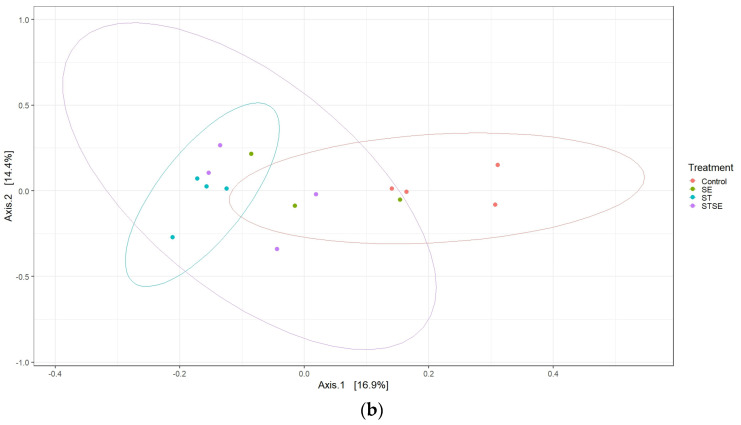
Beta diversity analysis on cecal microbiotas in *Salmonella* vaccinated and unvaccinated broiler chickens revealed that the live vaccines generally had a significant effect on chickens cecal microbiotas (*p* = 0.016) (**a**). Furthermore, the vaccines AviPro^®^ Salmonella Vac T and AviPro^®^ Salmonella DUO, but not the AviPro^®^ Salmonella Vac E, significantly altered the cecal microbiota compositions (*p* = 0.024), as compared with unvaccinated control chickens (**b**): (**a**) Beta diversity analysis per treatment type (vaccination versus unvaccinated controls); (**b**) Beta diversity analysis comparing the different *Salmonella* vaccines treated chickens and unvaccinated control chickens. The β-diversity was visualized using principal coordinate analysis (PCoA) plots, based on the Bray–Curtis dissimilarity indices. The adonis2 permutation multivariate ANOVA (PERMANOVA) was performed to analyze the general vaccination as well as different vaccine types’ influences on chicken cecal microbiotas. SE = AviPro^®^ Salmonella Vac E; ST = AviPro^®^ Salmonella Vac T; STSE = AviPro^®^ Salmonella DUO.

First, the live attenuated *Salmonella* vaccines in general and whether vaccination affected the cecal microbiota composition compared with the unvaccinated chickens were investigated. It can be seen (Figure 5a) that the vaccinated and unvaccinated chickens clustered differently from each other, suggesting that the treatments (live attenuated *Salmonella* vaccines) had a significant effect (*p* = 0.016) on the cecal microbiota.

Then, we compared the individual live *Salmonella* vaccines’ effect on cecal microbiota variability against the unvaccinated chickens. As indicated by the clustering into different groups (Figure 5b), our data suggest that the AviPro^®^ Salmonella Vac T and AviPro^®^ Salmonella DUO, but not the AviPro^®^ Salmonella Vac E vaccine, had a significant effect (*p* = 0.024) on the variability of the chicken cecal microbiota.

### 3.5. Cecal Microbiota Taxa Abundances

**Figure 6:**
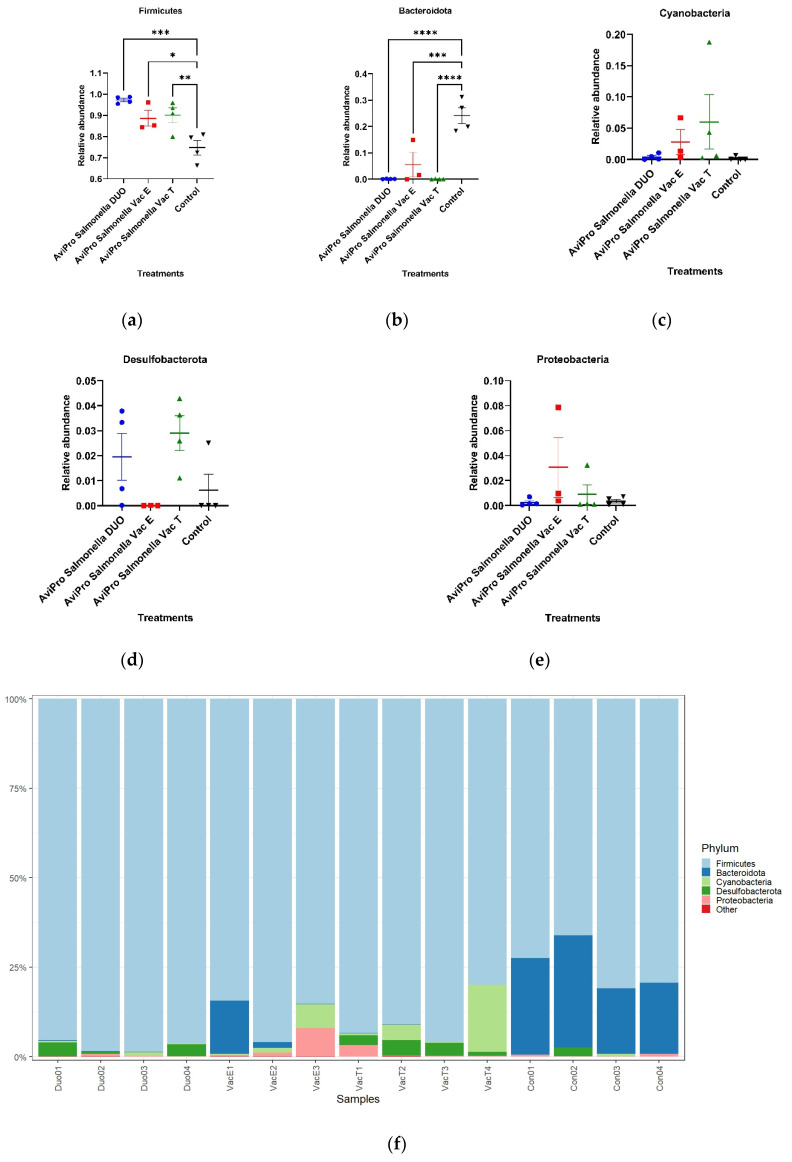
Chicken cecal microbiota relative abundances at the phylum level. Firmicutes was dominant in all vaccinated and unvaccinated chickens, but significantly higher abundances (*p* = 0.0006, 0.0267 and 0.0093, respectively) were revealed in vaccinated chickens and in the order AviPro^®^ Salmonella DUO > AviPro^®^ Salmonella Vac T > AviPro^®^ Salmonella Vac E > unvaccinated. The relative abundance for the phylum Bacteroidota was significantly higher in unvaccinated chickens as compared with the AviPro^®^ Salmonella DUO (*p* = 0.0001), AviPro^®^ Salmonella Vac E (*p* = 0.0008) and AviPro^®^ Salmonella Vac T (*p* = 0.0001). The relative abundances of other phyla in the vaccinated and control chickens were not significantly different (*p* values > 0.05): (**a**) Firmicutes relative abundance; (**b**) Bacteroidota relative abundance; (**c**) Cyanobacteria relative abundance; (**d**) Desulfobacterota relative abundance; (**e**) Proteobacteria relative abundance; (**f**) Phyla distribution and abundances in vaccinated and unvaccinated control chickens. Con01, Con02, Con03 and Con04 are the samples derived from the unvaccinated control chickens; VacT1, VacT2, VacT3 and VacT4 represent the samples from the live attenuated *Salmonella* Typhimurium (AviPro^®^ Salmonella Vac T) vaccinated chickens; VacE1, VacE2 and VacE3 are the samples from the live attenuated *Salmonella* Enteritidis (AviPro^®^ Salmonella Vac E) vaccinated chickens; Duo01, Duo02, Duo03 and Duo04 represent the samples derived from the AviPro^®^ Salmonella DUO vaccine (*Salmonella* Typhimurium + *Salmonella* Enteritidis) chickens. * = *p* ≤ 0.05; ** = *p* ≤ 0.01; *** = *p* ≤ 0.001; **** = *p* ≤ 0.0001.

To understand the influence of live *Salmonella* vaccines on chicken cecal microbiota taxa, we investigated their composition, distribution, and relative abundances in vaccinated and unvaccinated chickens. At the phylum level (Figure 6f), our data reveal that Firmicutes was dominant in all vaccinated and unvaccinated broiler chickens. Nevertheless, vaccinated chickens showed significantly higher abundances (*p* = 0.0006, 0.0267 and 0.0093, respectively) for this phylum compared with the unvaccinated controls (Figure 6a), and in the order AviPro^®^ Salmonella DUO (97.23%) > AviPro^®^ Salmonella Vac T (90.05%) > AviPro^®^ Salmonella Vac E (88.54%) > unvaccinated (74.72%).

The next dominant phylum was Bacteroidota, mainly found in unvaccinated chickens (24.11%) and the AviPro^®^ Salmonella Vac E (5.49%) vaccinated chickens. The lowest abundance for this phylum was, however, observed in the AviPro^®^ Salmonella Vac T (0.06%) and AviPro^®^ Salmonella DUO (0.08%) groups. The relative abundance for this phylum was significantly higher in unvaccinated chickens as compared with the AviPro^®^ Salmonella DUO (*p* = 0.0001), AviPro^®^ Salmonella Vac E (*p* = 0.0008) and AviPro^®^ Salmonella Vac T (*p* = 0.0001), Figure 6b.

Cyanobacteria was another abundant phylum and appeared almost exclusively in the vaccinated chickens. Even so, the AviPro^®^ Salmonella Vac T vaccinated chickens showed a relatively higher abundance (5.97%) compared with the AviPro^®^ Salmonella Vac E (2.77%) and the AviPro^®^ Salmonella DUO (0.40%). In unvaccinated chickens, the phylum Cyanobacteria was found at the lowest abundance (0.16%). Despite the observed visual trend, the relative abundances in the treatment groups were not significantly different (*p* values > 0.05) (Figure 6c). 

Proteobacteria was the other abundant phylum identified and it was common among vaccinated and unvaccinated chickens. However, the abundance was higher in the AviPro^®^ Salmonella Vac E (3.07%) and AviPro^®^ Salmonella Vac T (0.91%) as compared with the AviPro^®^ Salmonella DUO (0.26%) and unvaccinated (0.37%) chickens. The observed phylum abundances in the vaccinated and control chickens were, however, not significant (*p* values > 0.05), as per Figure 6e.

The phylum Desulfobacterota (Figure 6d) also appeared to be most common in the chickens that received the AviPro^®^ Salmonella Vac T and AviPro^®^ Salmonella DUO vaccines, with mean relative abundances of 2.91% and 1.95%, respectively. This phylum also appeared in the unvaccinated chickens but at low abundance (0.63%). Interestingly, this phylum was not identified in the AviPro^®^ Salmonella Vac E chickens. Nevertheless, the phylum abundances in the control and vaccinated chickens were not significantly different (*p* values > 0.05).

**Figure 7:**
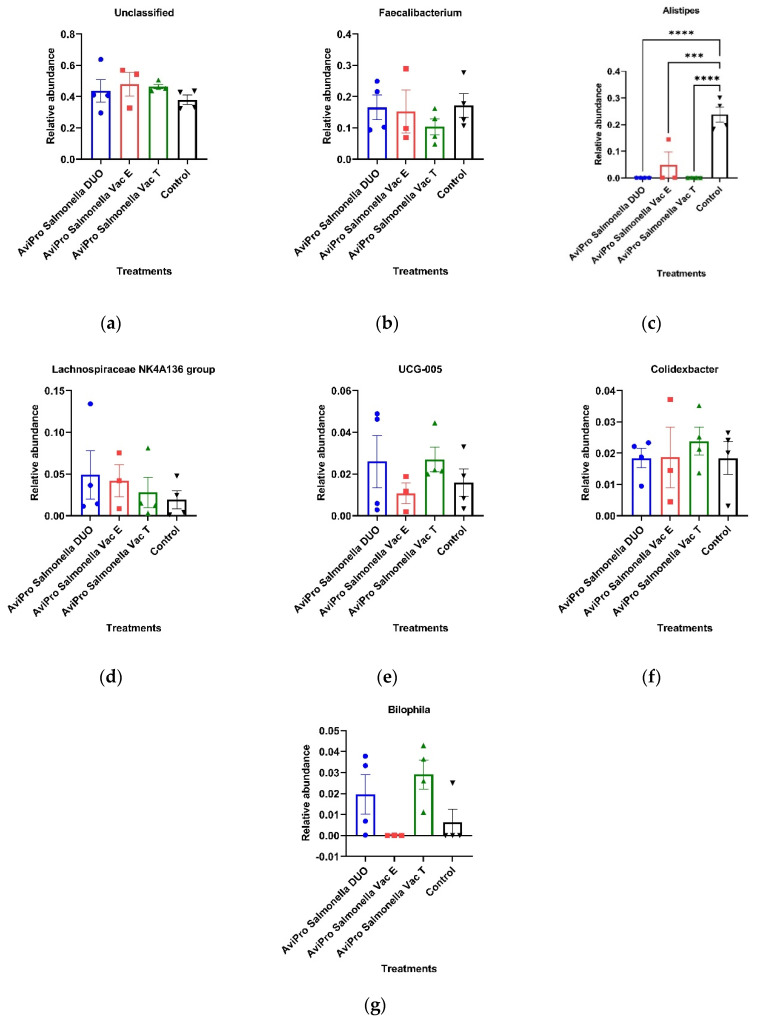

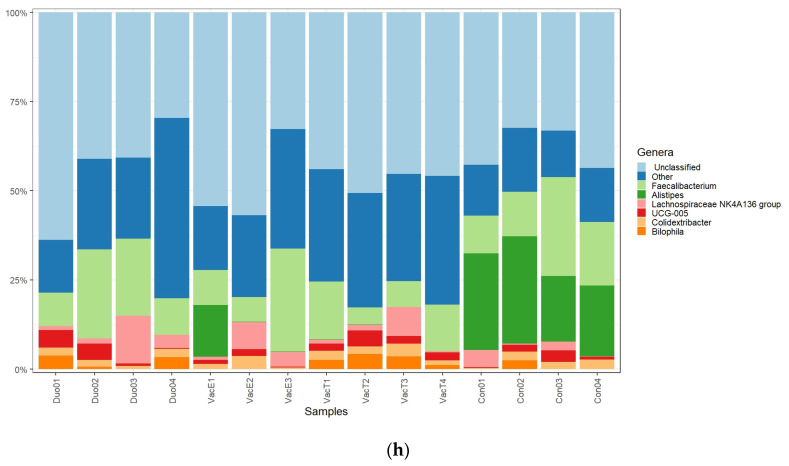
Chicken cecal microbiota relative abundances at the genus level. The genus *Alistipes* was mostly common in the unvaccinated control and at significantly higher abundances compared with the AviPro^®^ Salmonella DUO (*p* = 0.0001), AviPro^®^ Salmonella Vac E (*p* = 0.0006) and AviPro^®^ Salmonella Vac T (*p* = 0.0001) chickens. The abundance of other genera in the vaccinated and control unvaccinated chickens were not significantly different (*p* values > 0.05); (**a**) Unclassified genera relative abundances; (**b**) *Faecalibacterium* relative abundance; (**c**) *Alistipes* relative abundance; (**d**) *Lachnospiraceae* NK4A136 group relative abundance; (**e**) UCG-005 relative abundance; (**f**) *Colidextribacter* relative abundance; (**g**) *Bilophila* relative abundance; (**h**) Genera distribution and abundances in vaccinated and unvaccinated control chickens. Con01, Con02, Con03 and Con04 are the samples derived from the unvaccinated control chickens; VacT1, VacT2, VacT3 and VacT4 represent the samples from the live attenuated *Salmonella* Typhimurium (AviPro^®^ Salmonella Vac T) vaccinated chickens; VacE1, VacE2 and VacE3 are the samples from the live attenuated *Salmonella* Enteritidis (AviPro^®^ Salmonella Vac E) vaccinated chickens; Duo01, Duo02, Duo03 and Duo04 represent the samples derived from the AviPro^®^ Salmonella DUO vaccine (*Salmonella* Typhimurium + *Salmonella* Enteritidis) chickens. *** = *p* ≤ 0.001; **** = *p* ≤ 0.0001.

The genus *Alistipes* was mostly common in the unvaccinated chickens, in which it appeared at the highest abundance (23.80%) compared with the vaccinated groups. In vaccinated chickens, *Alistipes* was also identified but at low abundances of 4.88% (AviPro^®^ Salmonella Vac E), 0.04% (AviPro^®^ Salmonella Vac T) and 0.01% (AviPro^®^ Salmonella DUO). The genus abundance was significantly higher in the unvaccinated control than in the AviPro^®^ Salmonella DUO (*p* = 0.0001), AviPro^®^ Salmonella Vac E (*p* = 0.0006) and AviPro^®^ Salmonella Vac T (*p* = 0.0001) chickens (Figure 7c).

*Faecalibacterium* comprised another genus which was common in both vaccinated and unvaccinated chickens, and with similar abundances. The relative abundances for the genus were, respectively, 10.33% (AviPro^®^ Salmonella Vac T), 15.22% (AviPro^®^ Salmonella Vac E), 16.53 (AviPro^®^ Salmonella DUO) and 17.15% (unvaccinated) chickens and were not significantly different (*p* values > 0.05) (Figure 7b). 

Similarly, the genera *Colidextribacter*, UCG-005 and *Lachnospiraceae* NK4A136 groups showed a common distribution among the vaccinated and unvaccinated chickens. The relative abundances for these genera were also similar in different treatment groups; *Colidextribacter* (1.87% in AviPro^®^ Salmonella Vac E, 2.38% in AviPro^®^ Salmonella Vac T, and 1.84 in both the AviPro^®^ Salmonella DUO and unvaccinated chickens), UCG-005 (1.08% in AviPro^®^ Salmonella Vac E, 1.59% in unvaccinated, 2.60% in the AviPro^®^ Salmonella DUO and 2.70% in AviPro^®^ Salmonella Vac T chickens) and *Lachnospiraceae* NK4A136 group (4.2% in AviPro^®^ Salmonella Vac E, 1.92% in unvaccinated, 4.90% in the AviPro^®^ Salmonella DUO and 2.78% in AviPro^®^ Salmonella Vac T chickens). The abundances for these genera were nonetheless not significantly different (*p* values > 0.05) as per Figure 7d–f. 

The genus *Bilophila* was mostly common in the AviPro^®^ Salmonella Vac T (2.91%) and AviPro^®^ Salmonella DUO (2.00%) vaccinated chickens. It was also identified in one of the unvaccinated chickens (0.63%) and in the AviPro^®^ Salmonella Vac E vaccinated chickens at the lowest abundance (0.01%). Nevertheless, the observed abundance differences for this genus were not significant (*p* values > 0.05) (Figure 7g).

## 4. Discussion

The ban on antibiotics use in many parts of the world due to the increasing problem of antibiotic resistance has resulted in an urgent search for non-antimicrobial alternatives. Various alternatives have been developed. These include vaccines, which are widely applied in the poultry industry to limit *Salmonella* contamination at the farm level. Nevertheless, the mechanisms of action for most antimicrobial alternatives are yet to be fully understood [10,11], especially in the host–microbiota–pathogen context. Both inactivated and live *Salmonella* vaccines are commercially available and used in poultry farms, but live vaccines are considered more protective due to their ability to induce both cellular and humoral immune responses. Live attenuated *Salmonella* vaccines are mostly orally administered in poultry, exposing them to a complex environment comprising the host immune factors and commensal gut microbiota. Being live bacteria, it is expected that the vaccine strains will need to colonize the gut and establish a niche, although for a limited period of time. The effect that live attenuated *Salmonella* vaccine has on the poultry gut microbiota is not so clear. In this study, we used three commercial live attenuated *Salmonella* vaccine strains for *S.* Typhimurium (AviPro^®^ Salmonella Vac T), *S.* Enteritidis (AviPro^®^ Salmonella Vac E) and *S.* Typhimurium + *S.* Enteritidis (AviPro^®^ Salmonella DUO) to study their influence on chicken cecal microbiota profiles. Chicks were orally exposed to the attenuated vaccines and ceca, cecal contents, and sera sampled 14 days post-vaccination for analysis of cecal immune related gene expression, *Salmonella*-specific antibodies (IgG and IgA), and microbiota population structure.

The effect of live *Salmonella* vaccines on cecal immune genes was studied by comparing the expression of the cytokines involved in type 1 (IL-18, TNFα), type 2 (IL-4, IL-10, IL-6) and type 3 (IL-17A, IL-8) immune responses. The avian β-defensin (AvBD1), a cationic antimicrobial peptide secreted by gut epithelial cells as an effector signature of IL-17 was also analyzed. Forkhead Box P3 (FoxP3) and inducible nitric oxide synthase (iNOS) were also included to study the regulatory T-cells (Tregs) levels (indicative of the non-inflammatory, tolerogenic state, important for persistent *Salmonella* colonization) and oxidative burst status (iNOS), respectively. In chickens, FoxP3, is a recently discovered member of the FOX proteins family and acts as a pivotal regulator in the regulatory pathway for biosynthesis and for the functioning of Treg cells [24]. 

Generally, it is expected that invasive live *Salmonella* vaccines should induce similar immune responses as their pathogenic counterparts. Our data show a significantly high IL-8 expression in the AviPro^®^ Salmonella Vac E vaccinated chickens (Figure 1b). Following this cytokine’s role in the recruitment of heterophils to the inflammatory site, it could be suggested that this vaccine induced higher inflammation compared with the other two, although this might need further confirmation of heterophils infiltration into the cecal tissues. Furthermore, an oral immunization with attenuated *Salmonella* vaccine strains during this study triggered an upregulation of some cecal immune genes in broiler chickens, although the fold changes in their expression were not significant. This was the case for the AviPro^®^ Salmonella Vac E (Figure 1b), but not for the AviPro^®^ Salmonella Vac T (Figure 1a) or the AviPro^®^ Salmonella DUO (Figure 1c) in which iNOS and IL-18, and IL-4, iNOS and AvBD1 were downregulated. This is partly in agreement with the findings by Carvajal et al. [32] and Pan et al. [33], in which IL-8 (CXCLi2), IFNγ, IL-6, and IL-2 expression were induced in ceca following an oral immunization of chickens with an attenuated recombinant *Salmonella enterica* serovar Typhimurium vaccine candidate. Additionally, a study by Kogut et al. (2016) reported highly induced IL-6 expression on day 1, followed by a decrease until day 14 post infection with *Salmonella* Enteritidis in chicken ceca. The IL-10 expression, on the other hand, increased towards day 14 post infection [3]. Although our study was not time-point designed as was the case for Kogut et al., we also observed (Figure 1b,c) similar trends of IL-10 and IL-6 expression at sampling (14 days since vaccination). 

The expression of IL-17A (Figure 1a–c) was not induced above the control level following an oral exposure to all the studied live *Salmonella* vaccines. Congruent to our observation, an oral immunization with the SPI-1 mutant *S.* Enteritidis triggered low expression of IL-17 and IL-22 in chicken ceca [34]. It has been proposed that the interaction between *Salmonella* and the chicken gut triggers immune responses that can be divided into three stages, depending on the duration of interaction since exposure. The stages are associated with changes not only of the inflammatory status but also metabolic reprogramming towards less responsiveness against the pathogen [2]. Considering the sample collection timing post immunization and our cecal immune related gene expression data, it can be suggested that the anti-inflammatory (stage 2) and homeostatic (stage 3) responses were mostly dominant, as also indicated by the IL-10, FoxP3, iNOS, IL-4, IL-17A expression profiles. 

Taken together, our data suggest that the live attenuated *Salmonella* vaccine AviPro^®^ Salmonella DUO induced a more anti-inflammatory cecal environment (IL-10) and Th1 responses (IL-18) both of which are crucial for limiting the contamination level with *Salmonella* in chickens. Furthermore, the tolerogenic environment (FoxP3 expression), necessary for persistent colonization of chickens with *Salmonella* was lower in the AviPro^®^ Salmonella DUO vaccinated chickens, which might be indicative of improved protection. 

To study the vaccine-induced humoral responses, whole *Salmonella*-specific antibodies were quantified and compared in vaccinated and unvaccinated chickens. Specifically, IgG and IgA, which are the most dominant chicken immunoglobulins in serum and gut mucosa, respectively, were measured by ELISA. Although not significantly, our data show that the AviPro^®^ Salmonella Vac T induced an increase in serum IgG above the control level (Figure 2a). On the other hand, the cecal IgA level in all vaccinated chickens were generally low and not different from unvaccinated controls (Figure 2a). This is in contrast with the findings by Matsui et al. [35], in which oral administration of a live virulence plasmid-cured *S.* Typhimurium vaccine candidate in mice induced high anti-*S.* Typhimurium IgA and IgG levels in serum, cecal homogenates, intestinal and lung lavage fluids and in bile as compared with unvaccinated mice. Moreover, higher antibodies were observed following three oral vaccination rounds than following two rounds or one. Similar findings were also reported by Bridge et al. [36] in their study using mice orally or intraperitoneally vaccinated with a live *Salmonella* vaccine candidate. Immunization increased *Salmonella*-specific IgG and sIgA as compared with the unvaccinated controls, 25–27 days post vaccination. Furthermore, similar trends of IgA and IgG in serum and fecal pellets of mice [37] or swine [38] immunized with live attenuated *Salmonella* vaccines have been reported. The low and non-significant IgG and IgA levels in vaccinated chickens observed in our study could partly be contributed by a single oral administration of the live *Salmonella* vaccines prior to sampling. Nevertheless, the single oral vaccination round performed in this study was according to the manufacturers’ recommendation for broiler chickens. It is also specified by the manufacturer that low seropositivity reactivity of individual birds in a flock are possible following oral vaccination with the AviPro^®^ Salmonella Vac T, AviPro^®^ Salmonella Vac T, and AviPro^®^ Salmonella DUO (AviPro^®^ Salmonella DUO user manual, 2020) [39], which could be another possible explanation for our antibody ELISA data. 

Next, the live *Salmonella* vaccine’s effect on chicken cecal microbiota was investigated. The chicken gut is inhabited by millions of commensal microbiota which vary in composition and abundances with gut section, age of the bird, feed, water, breed, environmental factors (hygiene or biosecurity levels), and the host immune factors. The highest abundance is found in ceca, the two blind pouches located at the posterior end of the chicken gut, consisting of up to 10^10^–10^11^/g cecal content [40]. Microbiota are known to play various roles in the chicken gut, which determine the bird’s health via symbiotic interactions. These include the digestion of non-fermentable fibers, making them available for uptake by the bird; the inducing of gut development [40]; and the promotion of the development, maturation, training and functioning of chicken immunity [41,42].

There are conflicting data on the effect of *Salmonella* infection on gut microbiota. A study by Mon et al. [43] showed that *S.* Enteritidis induced a reduction in microbiota diversity in chicken, with members of the *Enterobacteriaceae* (Proteobacteria) family being dominant while the *Lachnospiraceae* family (particularly butyrate producers) and *Ruminococcus* were significantly reduced and negatively correlated with the *Enterobacteriaceae* family expansion due to competitive interaction between the two taxa, 7 days post infection. Early (2 days) post infection, Proteobacteria phylum expansion resulted in Firmicutes reduction. Similarly, a study by Khan and Chousalkar [44] reported reduced abundances in the genera Lactobacillus, *Faecalibacterium*, Bifidobacterium, *Alistipes* and Butyricimonas following an infection with virulent *S.* Typhimurium while increasing the Butyricicoccus, Oscillibacter and Eryscipelatoclostridium genera abundances.

On the other hand, Videnska et al. [45] reported a minor effect on microbiota alteration upon *Salmonella* infection in chickens. *Enterobacteriaceae* and *Ruminococcaceae* were shown to both increase in *S.* Enteritidis-infected chickens as compared with uninfected chickens, although this was not statistically significant. Likewise, the study by Zeng et al. [46] showed that at day 0 post infection with *S.* Enteritidis, the family *Enterobacteriaceae* dominated the chicken ceca. Ten days later, the microbiota were more diverse and stabilized, consisting of *Ruminococcaceae* (Firmicutes), *Enterobacteriaceae* (Proteobacteria), *Lachnospiraceae* (Firmicutes) and *Clostridiaceae* (Firmicutes). Overall, *S.* Enteritidis infection in 2-day old chickens did not significantly alter the cecal microbiota structure.

Looking into the influence of live attenuated *Salmonella* vaccines on cecal microbiotas, our data show that variability within samples (α-diversity measure) was only marginally significant in the AviPro^®^ Salmonella Vac T vaccinated chickens (Figure 4c). The other two vaccines (AviPro^®^ Salmonella DUO and AviPro^®^ Salmonella Vac E), however, did not indicate a significant influence on cecal microbiota variability (Figure 4a,b).

Although there were no differences in alpha diversity, interesting effects were observed for the β-diversity. Data from this study show that live vaccines in general had a significant influence on the cecal microbiota shaping, as suggested by the different clustering between vaccine and control chicken samples (Figure 5a). Next, we indicated that, individually, the AviPro^®^ Salmonella Vac T and AviPro^®^ Salmonella DUO, but not the AviPro^®^ Salmonella Vac E vaccine, had significant effects on the chicken cecal microbiota variability (Figure 5b).

At the phylum level, the current study revealed high Firmicutes abundance in broiler chickens irrespective of their vaccination status with the live attenuated *Salmonella* vaccines. The dominance of Firmicutes in broilers ceca observed here has also been reported by Oakley et al. [21], and that this was almost an exclusive phylum in broiler chickens older than a week. Similar results by Qi et al. [47], also showed that Firmicutes and Bacteroidota were the most dominant broiler cecal taxa at the phylum level. Nevertheless, our study showed that the live attenuated *Salmonella* vaccines significantly increased the Firmicutes relative abundances as compared with unvaccinated chickens (Figure 6). Contrary to our data, Park et al. [17] reported the highest abundance for Firmicutes in unvaccinated broiler chickens and the oral challenge with live attenuated *Salmonella* Typhimurium vaccine candidates (PBAD-mviN *S.* Typhimurium UK-1 and ΔΔ*metRmetD S.* Typhimurium UK-1) did not significantly alter this trend. A decrease in Firmicutes abundances has also been reported in layer chickens [48]. Unlike our study, however, the findings by Joat and colleagues followed an intramuscular injection with a wild-type *Salmonella* Typhimurium UK-1 strain.

Consistent with our observations at phylum level, most high abundant genera identified were also members of the Firmicutes. The genus *Faecalibacterium* is a well-known abundant cecal genus of the phylum Firmicutes [49] and with well-known commensal benefits to chickens. Some *Faecalibacterium* members have been described to have anti-inflammatory properties in human and mice [50]. As shown in Figure 7b, our data suggest that the different live *Salmonella* vaccines used in this study did not significantly influence this genus, which is in agreement with the previous report by Park et al. [17].

Similarly, the genera *Colidextribacter*, UCG-005, and the *Lachnospiraceae* NK4A136 group, which also all belong to the phylum Firmicutes showed a common distribution among the vaccinated and unvaccinated chickens. Additionally, the relative abundances for these genera were not significantly different among the treatment groups (Figure 7d–f). The findings by Park et al. [17], contrary to ours, however, showed an increase in *Lachnospiraceae* abundances in broiler chickens challenged with a PBAD-mviN *S.* Typhimurium UK-1 vaccine candidate as compared with the unvaccinated. This could be contributed by the difference in age at sampling (16 days) in the current study, as compared with six weeks in the study by Park and colleagues. Generally, the enhanced broiler cecal Firmicutes abundances by live *Salmonella* vaccines can be considered an improvement in protection and health following the phylum members’ roles in protecting the host against pathogens and complex carbohydrates degradation [50].

The phylum Bacteroidota, was more represented in the unvaccinated than in the vaccinated chickens (Figure 6f). Our data, thus, suggest that the live vaccines used significantly reduced the abundance of this phylum (Figure 6b). Some Bacteroidota genera, such as *Alistipes*, are known to be short chain fatty acid (SCFA) producers from indigestible fiber fermentation and hence have an anti-inflammatory role in the animal gut and can considered beneficial to the host [51,52]. A similar trend was also observed at the genus level, in which *Alistipes* was mostly common in the unvaccinated chickens for which it appeared at the highest abundance compared with the vaccinated groups (Figure 7). In line with our data, Orso et al. [53] reported a decrease in Bacteroidetes and SCFAs producing genera abundances following a live coccidiosis vaccine in chickens as compared with the unvaccinated control.

The present study also identified Cyanobacteria, Proteobacteria and Desulfobacterota among the abundant phyla. Congruent to our data, Cyanobacteria and Proteobacteria have also been reported by Orso et al. [53], as next to Firmicutes and Bacteroidetes in abundances for broiler chickens. Additionally, in their work, an increased Proteobacteria abundance following a live vaccine against coccidiosis is reported. Proteobacteria are mostly associated with the generation of an inflammatory environment in the gut leading to dysbiosis, following their fermentative metabolism which mostly favors pathogenic bacteria [51] such as *Salmonella*. This is contradictory to the vaccination purpose in *Salmonella* (a Proteobacteria member) control programs. Our findings are in agreement with those of Orso et al. (2021), but the high abundances of Proteobacteria in the vaccinated chickens observed in the current study (although not significant) could be explained by the live attenuated *Salmonella* vaccines in the ceca, as the sampling was performed within the vaccines shedding window (up to 28 days post vaccination).

Our data are partly in agreement with the findings of Park et al. [17] in which the live *Salmonella* Typhimurium vaccine candidates affected only the presence of some cecal microbiota without influencing their overall relative abundances. However, here, we also observed that some taxa relative abundances were significantly altered with the vaccination status, and this was the case for all the vaccines used in this study (Figure 6 and Figure 7).

## 5. Conclusions

Our data reveal that oral immunization with live attenuated *Salmonella* vaccines had a significant influence on the cecal microbiota variability in broiler chickens. Furthermore, we showed that different live vaccines could significantly affect the microbiota diversity and abundances differently. Following the roles that different commensal microbiota have in the gut, the induced microbiotas composition changes might in turn alter the chicken’s immune barrier function against pathogenic bacteria and overall health and productivity of the chicken. This knowledge might, thus, be useful when selecting live vaccines to use, particularly in integrated farming, where vaccination combined with the use of other microbiome influencers such as pro-and prebiotics is required. Further investigation on the direct correlation between gut immune-related gene expression and the cecal microbiota profiles and their functions would give more insights into how these interact with and shape one another. This would contribute to the design of improved prophylactic and therapeutic measures against *Salmonella enterica*, reducing poultry contamination and, thus, enhancing productivity and human food safety.

## Figures and Tables

**Table 1:** Primer and probe sequences used for qPCR of the selected chicken cecal immune genes.

Primer/Probe	Sequence (5′-3′)	Accession No.	Source
AvBD1 fwd	CCTCCTCCTGGCCCAGG	NM_204993.1	This study
AvBD1 rev	GCATTTCCCACTGATGAGAGTGAGG		This study
AvBD1 probe	(FAM)-CTGCAGGATCCTCCCAGGCTCTAGGAAGG-(ZEN/3′IBFQ)		This study
TNFα fwd	GCTGTTCTATGACCGCCCAGTT	NM_204267	[19]
TNFα rev	AACAACCAGCTATGCACCCCA		[19]
TNFα probe	(FAM)-CCTTCCTGTAACCAGATGATCGTGACACGTCTCTGC-(ZEN/3′IBFQ)		[19]
IL-8 fwd	GCTGCTCTGTCGCAAGGTAGG	DQ393272.2	This study
IL-8 rev	CAGGGAGCAGTGGGGTCC		This study
IL-8 probe	(FAM)-CGCTGGTAAAGATGGGGAATGAGCTGCGGT-(ZEN/3′IBFQ)		This study
IL-6 fwd	GCTCGCCGGCTTCGA	AJ309540	[20]
IL-6 rev	GGTAGGTCTGAAAGGCGAACAG		[20]
IL-6 probe	(FAM)-AGGAGAAATGCCTGACGAAGCTCTCCA-(ZEN/3′IBFQ)		[20]
IL-10 fwd	CATGCTGCTGGGCCTGAA	NM_001004414.2	[21]
IL-10 rev	CGTCTCCTTGATCTGCTTGATG		[21]
IL-10 probe	(FAM)-CGACGATGCGGCGCTGTCA-(ZEN/3′IBFQ)		[21]
IL-18 fwd	AGGTGAAATCTGGCAGTGGAAT	NM_204608.2	[21]
IL-18 rev	ACCTGGACGCTGAATGCAA		[21]
IL-18 probe	(FAM)-CCGCGCCTTCAGCAGGGATG-(ZEN/3′IBFQ)		[21]
IL-17A fwd	CATGGGATTACAGGATCGATGA	NM_204460	[22]
IL-17A rev	GCGGCACTGGGCATCA		[22]
IL-17A probe	(FAM)-ACAACCGCTTCCCCCGCTTGG-(ZEN/3′IBFQ)		[22]
iNOS fwd	AGGCCAAACATCCTGGAGGTC	U46504	[23]
iNOS rev	TCATAGAGACGCTGCTGCCAG		[23]
iNOS probe	(FAM)-CTGGAAGAGTTTCCTTCTGCTGAAGTCTCAACAG-(ZEN/3′IBFQ)		This study
FoxP3 fwd	AGTACGCCACAACCTGAGCCT	MT133687	[24]
FoxP3 rev	TTGGGGTCCTCTCAGCTCCGT		[24]
FoxP3 probe	(FAM)-TGCGGGTGGAGAACGTACGTGGG-(ZEN/3′IBFQ)		[24]
IL-4 fwd	AACATGCGTCAGCTCCTGAAT	AJ621735	[25]
IL-4 rev	TCTGCTAGGAACTTCTCCATTGAA		[25]
IL-4 probe	(FAM)-AGCAGCACCTCCCTCAAGGCACC-(ZEN/3′IBFQ)		[25]
GAPDH fwd	GTGGTGCTAAGCGTGTTATCATC	NM_204305	[26]
GAPDH rev	GGCAGCACCTCTGCCATC		[26]
GAPDH probe	(FAM)-CCCTCAGCTGATGCCCCCATGTTTGTGA-(ZEN/3′IBFQ)		This study

AvBD1 = avian β-defensin 1; TNFα = tumor necrotic factor α (also known as LITAF = lipopolysaccharide-induced TNF Factor); IL-8 = interleukin 8 (CXCLi2 = avian functional homology of mammalian IL-8); IL-6 = interleukin 6; IL-10 = interleukin 10; IL-18 = interleukin 18; IL-4 = interleukin 4; IL-17A = interleukin 17A; iNOS = inducible nitric oxide synthase; FoxP3 = Forkhead Box P3; GAPDH = glyceraldehyde 3-phosphate dehydrogenase. Fwd = forward primer; rev = reverse primer.

## Data Availability

The 16S rRNA raw sequence reads generated during this study have been deposited into the NCBI Sequence Read Archive (SRA) database and are publicly available via accession number PRJNA982296. All other data are also available on request from the corresponding author.
